# Footwear selection in an elderly population in relation to falls

**DOI:** 10.1186/1757-1146-6-S1-O10

**Published:** 2013-05-31

**Authors:** Annette Davis, Terrence Haines

**Affiliations:** 1 Southern Health Allied Health Research Department, Kingston Centre, Cheltenham, 3192, Victoria, Australia; 2 NHMRC Partnership Projects ID 546282; 3 Department of Health, Melbourne, Victoria, Australia

## Background

The NHMRC Partnership Project “Evidence based targeting of state wide strategies for preventing falls among community dwelling older people in Victoria” is a five component project examining the uptake of falls prevention strategies to better understand issues that might impact on the health and well-being of older adults in our community. From this data, information regarding drivers of footwear selection was determined and that is the focus of this study.

## Methods

Cross-sectional population based telephone survey of 394 men and women 70 years and over living in the community in Victoria. The survey was conducted over December 2010 to January 2011 and in a second wave between January 2012 to March 2012. Questions were asked regarding footwear worn indoors and outdoors and the reasons for this selection.

## Results

The choice of footwear worn indoors was overwhelmingly enclosed slippers with comfort as the reason for this selection. The outdoor footwear of choice was walking shoes with comfort once again the main driver for selection.

## Conclusion

Understanding footwear selection of the elderly may assist health care providers when recommending falls strategies for this at risk population.

